# Enhancing Home Blood Pressure Management: Implementation of the Sacubitril/Valsartan Treatment in Practical Clinical Settings

**DOI:** 10.31662/jmaj.2024-0262

**Published:** 2025-01-31

**Authors:** Tsugiyoshi Yamazaki

**Affiliations:** 1 Department of Cardiology, Tachibanadai Clinic, Yokohama, Japan

**Keywords:** Sacubitril/valsartan, Angiotensin receptor neprilysin inhibitor, Hypertension, Home blood pressure management, Blood pressure target achievement rate

## Abstract

**Introduction:**

Although the importance of home blood pressure (BP) management has been widely reported, the achievement rate of home BP targets remains low in Japan. Sacubitril/valsartan is a novel antihypertensive agent with potent antihypertensive effects. Despite its theoretical advantages, the real-world clinical application of sacubitril/valsartan in optimizing home BP management remains underexplored. The aim of this study was to evaluate the effect of switching from azilsartan treatment to sacubitril/valsartan treatment on the achievement of home BP targets and to refine hypertension management strategies in practical clinical settings.

**Methods:**

A cohort of 55 patients, with a mean morning home systolic BP of 135 mmHg or more was enrolled for an 8-week treatment phase with azilsartan and calcium-channel blockers. Morning BP, pulse rate (PR), estimated glomerular filtration rate, and B-type natriuretic peptide, serum potassium, serum uric acid (UA), and hemoglobin A1c levels were assessed at baseline and then at 8, 24, and 48 weeks after switching from 20 mg azilsartan to 200 mg sacubitril/valsartan.

**Results:**

At 48 weeks after switching to sacubitril/valsartan, there was a 60% increase in the rate of attainment of home systolic BP targets. Sacubitril/valsartan significantly reduced the mean systolic BP (from 143.6 ± 7.0 mmHg to 131.4 ± 8.7 mmHg), diastolic BP (from 86.9 ± 12.3 mmHg to 80.2 ± 10.7 mmHg), PR (from 74.8 ± 11.0 bpm to 72.1 ± 10.1 bpm), and serum UA (from 5.9 ± 1.1 mg/dL to 5.5 ± 0.9 mg/dL) within the first 8 weeks (all p < 0.01). These effects were maintained for 48 weeks.

**Conclusions:**

The switch from azilsartan to sacubitril/valsartan treatment resulted in a significant improvement in the achievement of home BP targets, which is consistent with our goal of refining hypertension management strategies in practical clinical settings.

## Introduction

Worldwide, hypertension is a major risk factor for cardiovascular disease, affecting an estimated 1.28 billion people, and its incidence is increasing ^(1)^. The increasing incidence underscores the urgency of improving hypertensive management strategies worldwide. In Japan, where hypertension is particularly prevalent, the guidelines of the Japanese Society of Hypertension (JSH2019) advocate strict targets for blood pressure (BP) at home: <125/75 mmHg for adults younger than 75 years and <135/85 mmHg for those 75 years and older ^(2)^. Despite these guidelines, only 21.3% of Japanese patients achieve these targets ^(3)^, indicating a substantial gap in effective home BP management.

Sacubitril/valsartan is an innovative angiotensin receptor neprilysin inhibitor that combines the neprilysin inhibitor sacubitril with the angiotensin II type I receptor blocker valsartan. This drug, which was initially approved for treating heart failure ^(4), (5)^, has demonstrated promising antihypertensive effects ^(6)^. In particular, in Japan, where high salt sensitivity and intake contribute to pronounced early morning and nocturnal BP spikes ^(7)^, the multiple antihypertensive mechanisms of sacubitril/valsartan, including improved urinary sodium excretion, represent a potential breakthrough in the treatment of uncontrolled morning hypertension.

Despite its theoretical advantages, the real-world clinical application of sacubitril/valsartan, especially in optimizing home BP management and its timely introduction for treating hypertension, remains underexplored. Therefore, this study aimed to fill this critical gap by investigating the effects of switching from azilsartan, an angiotensin II receptor blocker, to sacubitril/valsartan, specifically focusing on the effects of this switch on the achievement of morning home BP targets in Japanese patients struggling to control hypertension with conventional therapy [i.e., azilsartan and calcium channel blockers (CCBs)]. This study directly aligns with our goal of improving home BP management and provides insight into the practical implementation of sacubitril/valsartan treatment in daily clinical practice.

## Materials and Methods

### Study design and treatment

This single-center, open-label, prospective interventional study was conducted at the Tachibanadai Clinic, Japan, from October 2021 to November 2022. The participants were switched from oral azilsartan 20 mg (once daily) to oral sacubitril/valsartan 200 mg (once daily), and their health outcomes were monitored over a 48-week period. Key measurements included morning home BP and pulse rate (PR), as well as other relevant health markers including plasma B-type natriuretic peptide (BNP) concentrations, serum creatinine concentrations, estimated glomerular filtration rate (eGFR), serum potassium concentrations, serum uric acid (UA) concentrations, and hemoglobin A1c (HbA1c) values. These parameters were assessed at baseline and again at 8, 24, and 48 weeks after switching to evaluate the long-term effect of sacubitril/valsartan treatment on home BP management. Ethical approval was obtained from the Independent Ethics Committee of Isseikai Medical Corporation, and all participants provided informed consent. This study was registered in the UMIN Clinical Trials Registry (UMIN000052604).

### Subjects

The study included patients with essential hypertension who had a morning home systolic BP ≥135 mm Hg despite treatment with 20 mg azilsartan daily and CCBs for at least 8 weeks. The exclusion criteria included morning systolic BP ≥160 mmHg, secondary hypertension, nonsinus rhythm, heart failure, apparent infection, myocardial infarction, and/or stroke within 30 days, current pregnancy, active malignancy, eGFR <30 ml/min/1.73 m^2^, and plasma BNP level ≥100 pg/ml.

### BP and PR measurements

For accurate home BP management, morning home BP was measured twice consecutively after urination within 1 h of awakening and before breakfast, medication, or caffeine intake. The average of these two readings was recorded as the daily BP, and the average of daily BP readings over 7 consecutive days was used for evaluation as home BP. PR was measured concurrently with BP, with the average of two readings used as the daily PR, and the average over 1 week used for evaluation. The target morning home systolic BP was defined as <125 mm Hg for patients younger than 75 years and <135 mm Hg for patients 75 years and older, according to the JSH2019 guidelines.

### Statistical analysis

The data were presented as the mean ± standard deviation for continuous variables and as percentages for categorical variables. Changes in health parameters before and after switching were analyzed using paired t -tests. All statistical analyses were performed using EZR (Saitama Medical Center, Jichi Medical University, Saitama, Japan) in R (The R Foundation for Statistical Computing, Vienna, Austria) ^(8)^. More precisely, we used a modified version of R commander, which were designed to add statistical functions frequently used in biostatistics. P values <0.05 were considered statistically significant.

## Results

### Patient demographics

This study included 55 patients with hypertension. The mean age of the participants was 68.2 years, and the majority (63.6%) were male. The baseline characteristics, including BP and other health indicators, are detailed in Table 1.

**Table 1.** Baseline Patient Characteristics.

	*n* = 55
Sex, male, n (%)	35 (63.6)
Age (years)	68.2 ± 12.3
<75years, n (%)	35 (63.6)
≥75years, n (%)	20 (36.4)
Weight (kg)	69.1 ± 14.0
Body mass index (kg/m^2^)	25.7 ± 4.5
Morning home SBP (mmHg)	143.6 ± 7.0
Morning home DBP (mmHg)	86.9 ± 12.3
Current smoker, n (%)	3 (5.5)
Other complications, n (%)	
Type 2 diabetes mellitus	5 (9.2)
Dyslipidemia	33 (60.0)
Hyperuricemia	18 (32.7)
Coronary artery disease	6 (10.9)
Stroke	3 (5.5)
Sleep apnea syndrome	8 (14.5)
Chronic kidney disease	10 (18.2)
Antihypertensive drugs, n (%)
Calcium channel blockers	55 (100)
Angiotensin receptor blockers	55 (100)
Angiotensin-converting enzyme inhibitors	0 (0)
Diuretics	2 (3.6)
β blockers	5 (9.1)
Mineralocorticoid receptor antagonists	7 (12.7)
Others	0 (0)

Data presented as n (%) or mean ± SD *SBP,* Systolic blood pressure; *DBP,* Diastolic blood pressure

### Efficacy

#### Home systolic BP target achievement rate

**Figure 1.**
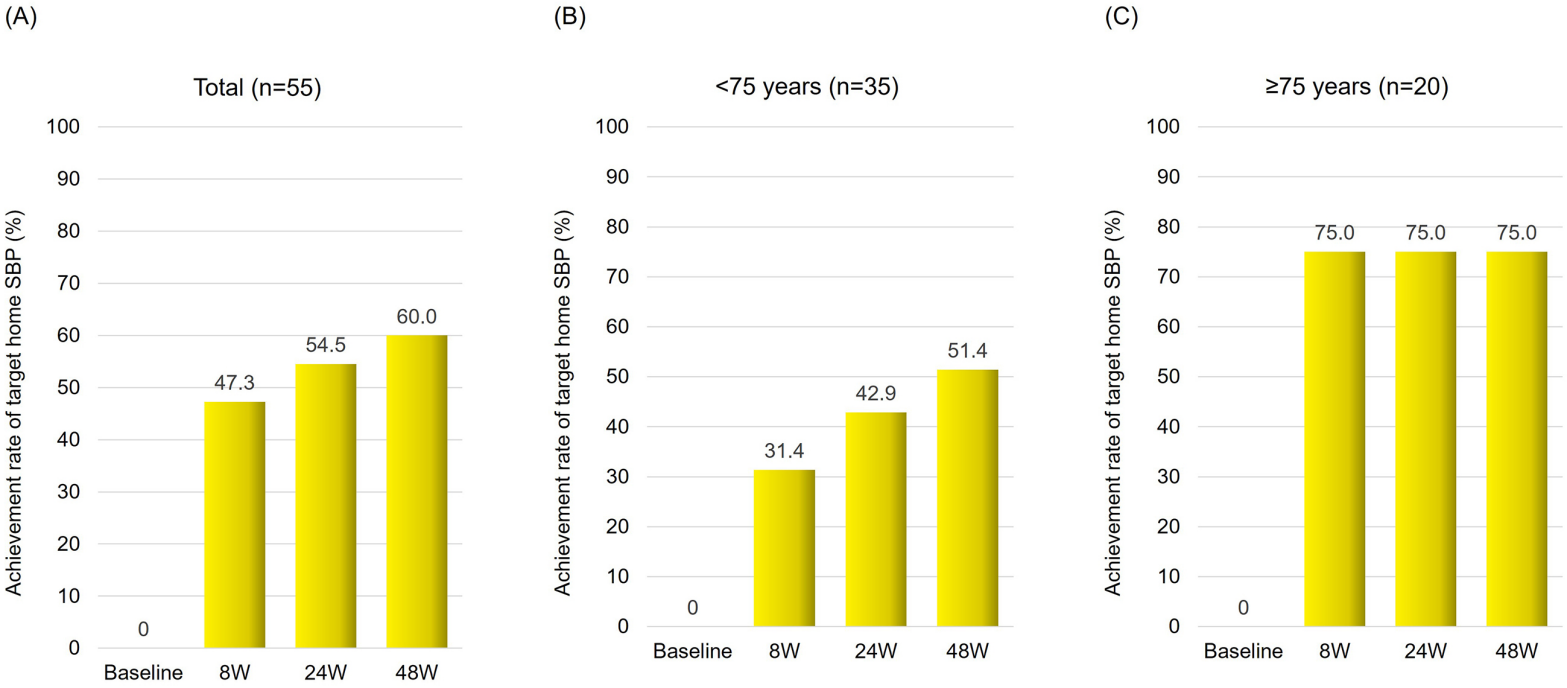
Achievement rate of target home systolic blood pressure after switching from azilsartan to sacubitril/valsartan Achievement rate of target home SBP after switching from azilsartan to sacubitril/valsartan treatment in the total population (A), and patients aged <75 years (B) and ≥75 years (C). SBP, systolic blood pressure.

The switch from azilsartan to sacubitril/valsartan treatment resulted in significant improvement in home systolic BP control. After 8 weeks, the target achievement rate increased by 47.3%, reaching 54.5% after 24 weeks and 60.0% after 48 weeks of treatment (Figure 1A). Notably, at 48 weeks, patients aged ≥75 years showed a greater rate of improvement (75.0%) compared to patients aged <75 years (51.4%) (Figure 1B and 1C).

#### BP-lowering effects

**Figure 2.**
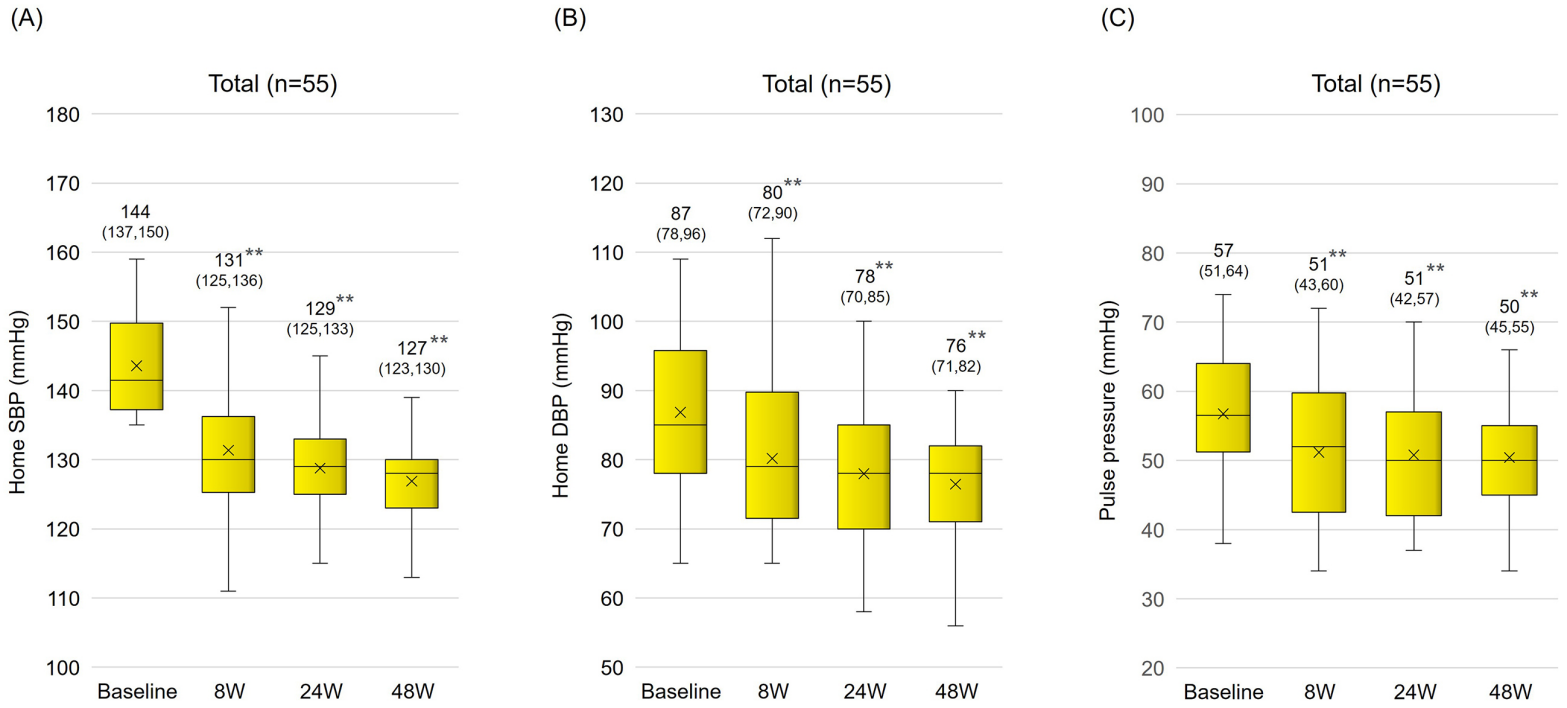
Changes in home blood pressure after switching from azilsartan to sacubitril/valsartan Changes in home SBP (A), DBP (B), and pulse pressure (C) after switching from azilsartan to sacubitril/valsartan in the total population. The numbers and numbers in brackets on each box plot indicate the mean and interquartile range, respectively. **p < 0.01 vs. baseline, paired t test. SBP, systolic blood pressure; DBP, diastolic blood pressure.

**Figure 3.**
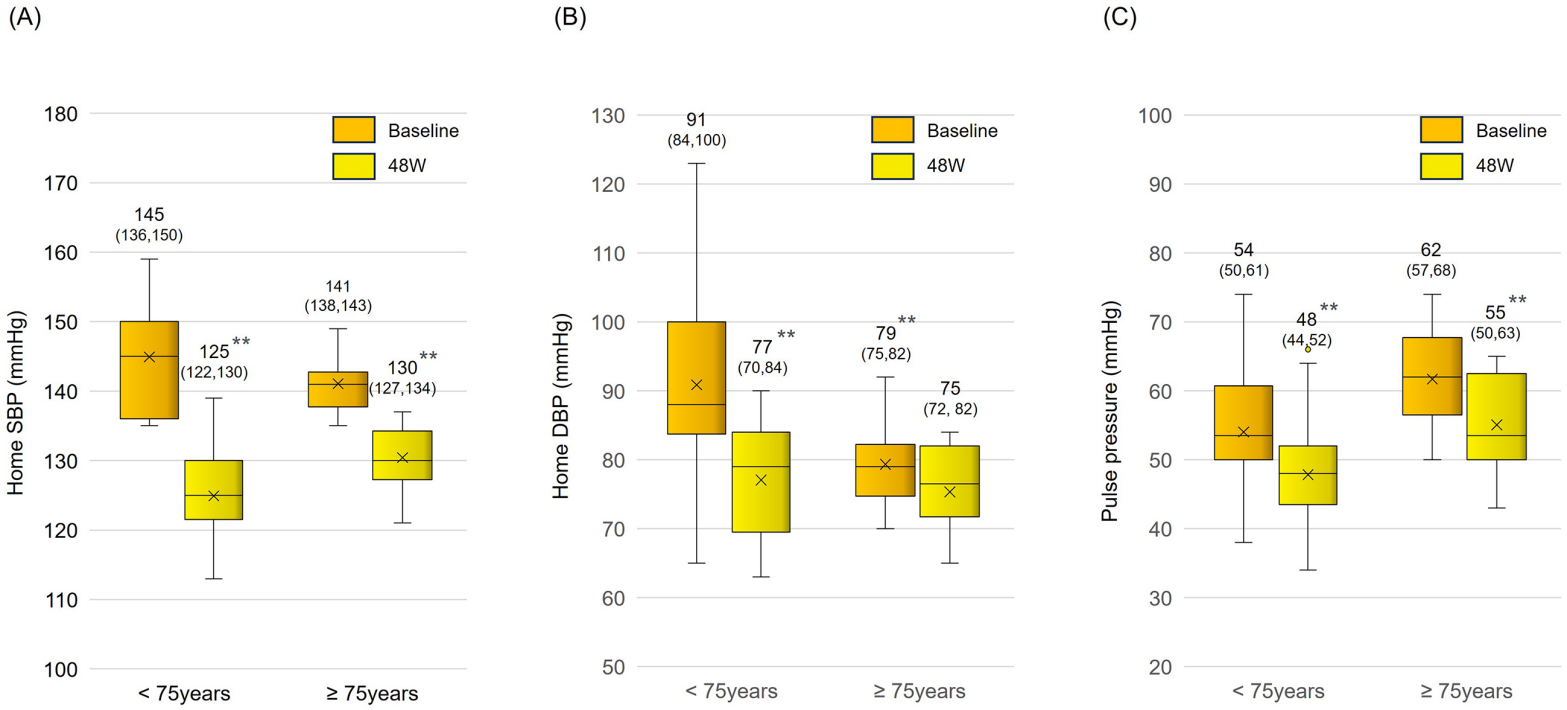
Changes in home blood pressure after switching from azilsartan to sacubitril/valsartan Changes in home SBP (A), DBP (B), and pulse pressure (C) after switching from azilsartan to sacubitril/valsartan in patients aged <75 years and ≥75 years. The numbers and numbers in brackets on each box plot indicate the mean and interquartile range, respectively. **p < 0.01 vs. baseline, paired t test. SBP, systolic blood pressure; DBP, diastolic blood pressure.

**Figure 4.**
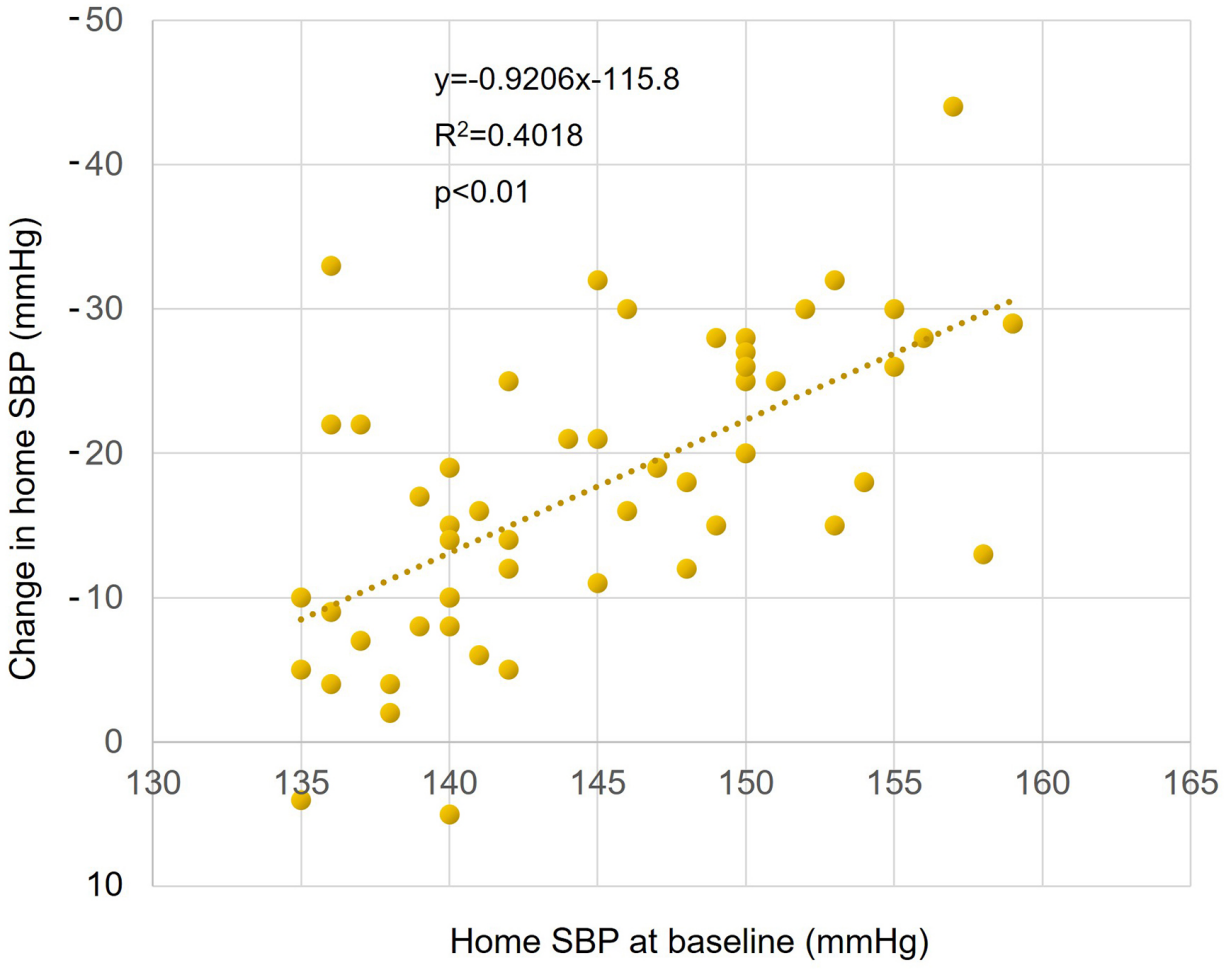
Correlation between baseline home systolic BP and change in home systolic BP after switching from azilsartan to sacubitril/valsartan treatment Correlation between baseline home SBP and change in baseline home SBP after switching from azilsartan to sacubitril/valsartan treatment in the total population. Pearson’s correlation coefficient (R^2^) is shown above the scatter plot. SBP, systolic blood pressure.

Sacubitril/valsartan treatment resulted in significantly reduced mean systolic BP (from 143.6 ± 7.0 mm Hg to 131.4 ± 8.7 mmHg) and diastolic BP (from 86.9 ± 12.3 mm Hg to 80.2 ± 10.7 mmHg) within the first 8 weeks. These reductions were maintained over a 48-week period (Figure 2A and 2B). The pulse pressure also significantly decreased (from 56.7 ± 10.0 mm Hg to 51.2 ± 9.5 mmHg), indicating a sustained antihypertensive effect (Figure 2C). In an age-specific analysis, in which subjects were classified into two age groups (<75 years old and ≥75 years old), a significant long-term reduction in BP and pulse pressure was also observed (Figure 3A, 3B and 3C). There was a significant positive correlation between systolic BP before switch and change in systolic BP (p < 0.01) (Figure 4).

#### Other health parameters

After 8 weeks, there was a significant reduction in the PR (from 74.8 ± 11.0 bpm to 72.1 ± 10.1 bpm), which further decreased to 69.5 ± 9.7 bpm by the end of the study. Serum UA levels also decreased significantly (from 5.9 ± 1.1 mg/dl to 5.5 ± 0.9 mg/dl). However, there were no significant changes in eGFR or serum potassium, creatinine, plasma BNP, and HbA1c levels (Table 2).

**Table 2.** Changes in Health Parameters after Switching from Azilsartan to Sacubitril/Valsartan.

	Baseline	48 week	P-value
Pulse rate (bpm)	74.8 ± 11.0	69.5 ± 9.7	<0.001
eGFRcreat (mL/min/1.73m^2^)	66.1 ± 14.5	65.4 ± 13.1	0.33
Serum potassium concentration (mEq/L)	4.3 ± 0.3	4.3 ± 0.3	0.39
Serum uric acid concentration (mg/dL)	5.9 ± 1.1	5.5 ± 1.0	<0.001
Plasma BNP concentration (pg/mL)	27.8 ± 21.2	28.9 ± 24.5	0.39
Hemoglobin A1c value (%)	5.8 ± 0.5	5.8 ± 0.5	0.42

Data are mean ± SD. P-value was determined using the paired t-test. *eGFRcreat*, creatinine-based estimate of glomerular filtration rate; *BNP*, brain natriuretic peptide

#### Safety profile

During the study, two patients discontinued sacubitril/valsartan because of the occurrence of mild adverse events including general malaise and increased urination with weight loss. Importantly, no significant safety concerns related to severe hypotension, renal dysfunction or electrolyte imbalances were noted.

## Discussion

### Improvement in home BP management with sacubitril/valsartan treatment

The results of this study underscore the pivotal role of sacubitril/valsartan treatment in improving home BP management in patients with hypertension inadequately controlled by conventional therapies. The switch from azilsartan to sacubitril/valsartan treatment resulted in a significant improvement in the achievement of home BP targets, aligning with our goal of refining hypertension management strategies in practical clinical settings.

### Sustained BP- lowering effects in a clinical context

Our results showed that sacubitril/valsartan not only effectively reduced morning home systolic and diastolic BP, but also maintained these reductions over a 48-week period. Given that hypertension is a major risk factor for cerebrovascular disease, sustained BP- lowering holds great promise in reducing the risk of cardiovascular events. This is particularly important considering the findings of a meta-analysis showing that a 10 mm Hg reduction in systolic BP can significantly reduce the risk of cardiovascular events and mortality ^(9), (10)^.

### Morning BP control: Addressing key risk factors

The emphasis on morning BP control is particularly relevant because morning BP is increasingly recognized as a critical risk factor for stroke and coronary heart disease ^(11), (12), (13), (14)^. The ability of sacubitril/valsartan to effectively control morning BP could be a major step forward in reducing these risks.

### Sacubitril/valsartan: A new approach for treating hypertension in Japan

With a significant proportion of Japanese patients struggling to achieve target BP levels, our study identified sacubitril/valsartan as a novel and effective treatment option. This is particularly important given the prevalence of inadequate hypertension treatment in Japan and the potential of sacubitril/valsartan to address this issue by simplifying treatment regimens and reducing the risk of polypharmacy.

### The role of natriuretic peptides in the treatment of salt-sensitive hypertension

The dual action of sacubitril/valsartan, which enhances the natriuretic peptide system while inhibiting the RAAS, offers a unique therapeutic approach, particularly for salt-sensitive hypertension, a common problem in Japan. Our results align with a growing body of evidence suggesting the efficacy of natriuretic peptides in managing conditions characterized by high salt sensitivity and intake ^(7), (15)^.

### Implications for managing PR and UA levels

The observed reductions in PR and serum UA levels further contribute to the cardiovascular protective effects of sacubitril/valsartan. These findings are particularly relevant in the context of the known associations between PR, hyperuricemia and increased cardiovascular risk ^(16), (17)^.

### Safety and tolerance in clinical practice

Sacubitril/valsartan therapy demonstrated a favorable safety profile in our study, with only minor adverse events leading to discontinuation. This aspect is crucial for ensuring patient adherence and the long-term efficacy of treatment.

### Limitations and future directions

Although our study provides valuable insights, it is not without limitations, including its single-center design and relatively small sample size. An important issue in the diagnosis and management of hypertension is the phenomenon of regression to the mean, that is, the convergence of high BP to lower values (and low BP to higher values) across repeated measurements. This effect is particularly relevant in studies in which participants with elevated BP are selected for intervention, as observed in the current study. Imai Y et al. (2001) highlighted the importance of home BP monitoring in detecting treatment effects despite potential regression to the mean ^(18)^, and recent evidence further suggests that regression to the mean is a significant consideration in interpreting BP trends in hypertensive populations ^(19)^. In our study, we selected patients with persistently high BP despite an 8-week treatment period with azilsartan and CCBs, thus controlling for transient BP variability. Following the switch to sacubitril/valsartan, the achieved BP reductions were maintained over 48 weeks, suggesting a stable treatment effect rather than a temporary shift due to regression to the mean. Nonetheless, this was a single-arm study without a control group; thus, a double-blind, randomized controlled trial is necessary to definitively separate the treatment effect from regression to the mean. In this study, there was no significant change in eGFR after the change to sacubitril valsartan, but proteinuria and albuminuria, which are important in the assessment of renal function, were not evaluated. Further evaluation of proteinuria and albuminuria is needed to assess the effect of sacubitril valsartan on renal function. Future research should include larger multicenter studies to validate these findings and explore the long-term effects of sacubitril/valsartan treatment on home BP management.

### Conclusion

In conclusion, the results of our study indicate that sacubitril/valsartan treatment is a promising option for improving home BP management in patients with uncontrolled hypertension. The implementation of this approach in real-world clinical practice could significantly improve hypertension treatment strategies, particularly in the Japanese context.

## Article Information

### Conflicts of Interest

Tsugiyoshi Yamazaki received honoraria from Novartis Pharma, Otsuka, and Mochida Pharmaceutical.

### Acknowledgement

I thank all the study participants and medical staff at the Tachibanadai Clinic.

### Author Contributions

Tsugiyoshi Yamazaki contributed to the design and implementation of the research, analysis of the results, and writing of the manuscript.

### Approval by Institutional Review Board (IRB)

All procedures conformed to the Declaration of Helsinki 2013. Ethical approval was obtained from the Independent Ethics Committee of Isseikai Medical Corporation on November 21, 2022 (approval number: 2022-03). The study is registered in the UMIN Clinical Trials Registry (UMIN000052604).

### Informed Consent

Written informed consent was obtained from all participants.
